# The first Brevinin-1 antimicrobial peptide with LPS-neutralizing and anti-inflammatory activities *in vitro* and *in vivo*

**DOI:** 10.3389/fmicb.2023.1102576

**Published:** 2023-03-03

**Authors:** Maolin Tian, Kai Wang, Yan Liang, Jinwei Chai, Jiena Wu, Haiyun Zhang, Xiaowen Huang, Xin Chen, Xueqing Xu

**Affiliations:** ^1^Guangdong Provincial Key Laboratory of New Drug Screening, School of Pharmaceutical Sciences, Southern Medical University, Guangzhou, China; ^2^Department of Critical Care Medicine, Zhujiang Hospital, Southern Medical University, Guangzhou, China; ^3^Department of Pulmonary and Critical Care Medicine, Zhujiang Hospital, Southern Medical University, Guangzhou, China; ^4^Department of Dermatology, Nanfang Hospital, Southern Medical University, Guangzhou, China

**Keywords:** antimicrobial peptide, amphibian, Brevinin-1, lipopolysaccharide, anti-inflammation

## Abstract

Antimicrobial peptide is one important component of the first protective barrier of organisms. They not only have potent antimicrobial activity which can protect the body from the invading pathogens, but also participate in the immune regulation of the body. In this study, a Brevinin-1 peptide named by Brevinin-1GHd was identified from *Hoplobatrachus rugulosus*, and the similarity of mature peptide sequence among Brevinin-1GHd, Brevinin-1HL and Brevinin-1GHa supported the close species relationship between *H. rugulosus*, *Hylarana latouchii* and *Hylarana guertheri*. Moreover, the secondary structure of Brevinin-1GHd was found to possess α-helical characteristics and high thermal stability. In addition, Brevinin-1GHd could bind to LPS with a Kd value of 6.49 ± 5.40 mM and suppress the release of TNF-α, NO, IL-6 and IL-1β by inactivation of MAPK signaling pathway in RAW 264.7 cells induced by LPS. Furtherly, Brevinin-1GHd had a significant inhibitory effect on acute edema development in the right paw of mice injected by carrageenan. Thus, the significant LPS-neutralizing and anti-inflammatory activities of Brevinin-1GHd were demonstrated in this study, which made it become the first Brevinin-1 family peptide with anti-inflammatory activity reported so far, and the biological activity of Brevinin-1GHd made it promising to be a novel therapeutic drug for infectious inflammation.

## 1. Introduction

Antimicrobial peptides (AMPs) make amphibians survive under the harsh and complex living environment with direct threat of abundant microorganisms and the infection hazard. More and more researchers focus on the functions and structure identification of amphibian AMPs (Xu and Lai, 2015; Annunziato and Costantino, 2020), which are reported to have antimicrobial, LPS-neutralization, immune regulation, anti-virus, anti-tumor, anti-oxidation, wound healing, promoting insulin release and other functions at present (Zhang et al., 2021). AMPs from the skin secretions of amphibians are extremely diverse and more than 100 families have been identified according to their sequence similarity (Xu and Lai, 2015). Brevinin family is one of the richest members, which is composed of two sub-families: Brevinin-1 and Brevinin-2 (Conlon, 2008; Nicolas and El Amri, 2009). The first Brevinin peptide was discovered in 1992 and about 350 Brevinin peptides have been found so far (Savelyeva et al., 2014). Most Brevinins shows broad-spectrum antibacterial and high hemolytic activity. In addition, these peptides are usually linear, amphiphilic and cationic and have a C-terminal Rana box (Cys-(Xaa) 5-Cys), which is bridged by a disulfide (Novkovic et al., 2012). Furtherly, although both Brevinin-1 and-2 generally have four unchanged residues, namely Ala^9^, Cys^18^, Lys^23^, Cys^24^ and Lys^7^, Cys^27^, Lys^28^, and Cys^33^, the amino acid sequences of Brevinin peptides are not conservative among different species, which makes Brevinin family rich in biological functions including antimicrobial activity, insulin releasing, and anti-cancer activities (Conlon et al., 2004, 2005). Noteworthy, immunoregulatory activity has been reported from the members from Brevinin-2 rather than Brevinin-1 (Popovic et al., 2012).

*Hoplobatrachus rugulosus* is the only species of *Hoplobatrachus* recorded in China (Frost, 2016). According to Chinese medical literature, “Northeast Animal Medicine,” *H. rugulosus* can be used as a medicine with the functions reducing swelling, detoxification and relieving cough (The Fourth Clinical College of Jilin Medical University, 1972). Although Tigerinin-1R and HR-CATH with antimicrobial activity are identified from *H. rugulosus* skin (Ojo et al., 2011; Chen et al., 2021), its other components remain stills indistinct. At present, a Brevinin-1 peptide, Brevinin-1GHd (Bre-1GHd in abbreviation) with LPS-neutralizing and anti-inflammatory activities *in vitro* and *in vivo*, was identified from the skin of *H. rugulosus*. It is so far the first report about Brevinin-1 peptide with anti-inflammatory activity, and this finding may be the beginning of studying the anti-inflammatory function in Brevinin-1 family peptides and provide a novel therapeutic drug for infectious inflammation.

## 2. Materials and methods

### 2.1. Animals and ethical statement

Adult *H. rugulosus* (*n* = 3) were purchased from various farms from Guangzhou, Guangdong province, China. The frogs were humanely euthanized with CO_2_ and cleaned with distilled water, and their dorsal skins were cut off and stored in liquid nitrogen. Mice from the Experimental Animal Center of Southern Medical University (Guangdong, China) were raised at 25°C with 12 h light/dark cycle and normal rodent rearing protocols. All experimental procedure related to animals were authorized by the Animal Care and Use Ethics Committee of Southern Medical University and strictly carried out in light of the guidelines of the Animal Care and Use Ethics Committee.

### 2.2. Brevinin-1GHd cDNA sequencing analysis

The cDNA sequence encoding Brevinin-1GHd from *H. rugulosus* was identified from the skin transcriptome (unpublished data). Molecular mass and theoretical isoelectric point were analyzed using ProtParam.^1^ Signal peptide was predicted with SignalP-6.0.^2^ The simulated secondary structure model of Brevinin-1GHd was predicted by trRosettaX-Single^3^ and visualized by PyMOL. The trRosetta algorithm built a three-dimensional structural model based on direct energy minimization (Du et al., 2021; Supplementary Figure S1). The Brevinin-1 family peptide sequences with high homology to Brevinin-1GHd were obtained by Basic Local Alignment Search Tool (BLAST) and were aligned using the ClustalW method. Phylogenetic Tree was constructed with MEGA X package^4^ by using the neighbor-joining method (Kumar et al., 2018).

### 2.3. Peptide synthesis

Brevinin-1GHd (FLGALFKVASKLVPAAICSISKKC) was synthesized and purified by GL Biochem Ltd. (Shanghai, China). The peptide was eluted with 46% acetonitrile solution at the flow rate of 1 ml/min with an inert silica gel ODS-SP column (Shimazu, Sumi, Japan) *via* HPLC. When the peptide purity was over 95% according to the ratio of different front areas, the peak was gathered and lyophilized, and further determined by MALDI-TOF mass spectrometry (Supplementary Figures S2, S3).

### 2.4. Circular dichroism analysis

Circular dichroism (CD) spectroscopy of Brevinin-1GHd was subjected using Chirascan plus ACD spectropolarimeter (Applied Photophysics Ltd., Leatherhead, United Kingdom) as previously described by us to estimate its secondary structure and interaction with LPS (L2880, *Escherichia coli* O55: B5, Sigma-Aldrich, St. Louis, Missouri, MO) (Wu et al., 2021). Shortly, Brevinin-1GHd at the final concentration of 50 μM was dissolved in SDS solution (0, 30, 60, 90, and 120 mM) or in 60 mM SDS solution before incubation at different temperatures (25, 37, 50, 70, and 90°C) for 1 h, and the CD spectrum was subsequently determined. To explore the binding of Brevinin-1GHd to LPS, 0.2 mg/ml of LPS was dissolved in H_2_O or 30 mM SDS solution, and then was mixed with 50 μM Brevinin-1GHd for 1 h at 25°C. Soon afterwards, the CD spectrum was determined. The binding of Brevinin-1GHd with LPS was identified by comparing the changes of spectrogram of each sample. CD data are presented as the mean residue ellipticity of three consecutive scans per sample in θ, deg.·cm^2^·dmol^−1^. The mean residue ellipticity (θ, deg.·cm^2^·dmol^−1^) is calculated by following equation: $\theta =\theta_{obs}/\left(10\times c\times l\times n\right)$, where θ_obs_ is the observed ellipticity (mdeg), c is the concentration (mol/L) of Brevinin-1GHd solution, l is the path length (cm), and n is the number of Brevinin-1GHd residues.

### 2.5. SPRi assay

The real-time binding reaction of Brevinin-1GHd with LPS was further measured with PlexArrayTM HTA100 system (Plexera LLC, Bothell, Washington, DC) and bare gold SPRi chip (Nanocapture gold chip, with a gold layer of 47.5 nm thickness) as previously described by us (Chai et al., 2021). Briefly, 2 mM Brevinin-1GHd was immobilized onto the surface of gold chips and LPS (1.25, 2.5, and 5 μM) as the analyte was flowed through the chip. The PLEXEA data analysis module and Prism 6.0 were used to analyze the data finally displayed as a curve.

### 2.6. Isothermal titration calorimetry assay

Interactions between Brevinin-1GHd and LPS were further determined with MicroCal PEAQ-ITC (Malvern; United Kingdom). Both Brevinin-1GHd and LPS powders were added to PBS solution to prepare stock solutions at concentrations of 0.5 mM and 50 μM, respectively. 280 μl of 50 μM LPS was applied into the sample cell with constant stirring speed of 250 rpm/s at 25°C before a 1.5 μl aliquot of Brevinin-1GHd at 0.5 mM was titrated into the sample cell. Instrument operation and data analysis were finished as previously described by us (Chai et al., 2021).

### 2.7. Membrane binding assay

Membrane binding assays were performed with Brevinin-1GHd as previously described with slight modification (Tian et al., 2021). Shortly, FITC-labeled Brevinin-1GHd was generated with FITC conjugation kit (Sangon Biotech, Shanghai, China) according to the manufacture’s manual. RAW 264.7 cells at a density of 1× 10^5^ cells/ml were incubated with different concentrations of FITC-labeled Brevinin-1GHd (0, 1.25, 2.5, 5 and 10 μM) at 37°C for 30 min. The unbound peptide was washed out with PBS for three times. The fluorescence intensity was measured with a FACscan flow cytometer (Becton Dickinson, United States).

### 2.8. Cytotoxicity assay

The cytotoxicity of Brevinin-1GHd toward RAW 264.7 cells was determined by cell counting kit-8 (CCK-8; Biosharp, China). RAW 264.7 macrophages at 2× 10^4^ cells/well were grown in 96-well plates containing DMEM medium with 10% FBS, 100 U/ml ampicillin plus 100 μg/ml streptomycin at 37°C. After adhesion, the cells were treated with different concentrations of Brevinin-1GHd (0, 1.25, 2.5, 5, 10 and 20 μM) for 24 h. The absorbance values of all wells at 450 nm was measured with microplate reader (Tecan Trading AG, Männedorf, Switzerland). All experiments were performed with at least three repeats.

### 2.9. Quantitative real-time PCR (qRT-PCR)

The cells were pre-treated with Brevinin-1GHd (1, 2, and 4 μM) for 30 min and were cultured with MDEM medium containing 100 ng/ml LPS for 8 h at 37°C. The cells lacking LPS and Brevinin-1GHd treatment were regarded as the negative control. Cells were lysed with trizol and isolated with chloroform, isopropanol to obtain total RNA. After the first-strand cDNA was synthesized by reverse transcription kit (Takara Company, Dalian, China) in light of the manufacturer’s manual, qRT-PCR was performed using an Applied Biosystems 7,500 instrument (Applied Biosystems, America) for estimation of mRNA expression of IL-1β, IL-6, TNF-α, and iNOS. qRT-PCR procedure and data analysis were performed as previously reported (Zeng et al., 2018). All experiments were performed with at least three repeats. The primer sequences used for the different tested genes are shown in the Supplementary Table S1.

### 2.10. No and inflammatory cytokine determination

RAW 264.7 cells at 2× 10^4^/well were grown in 96-well plates overnight and then were treated with Brevinin-1GHd (1, 2, and 4 μM) or PBS for 1 h before incubation with 100 ng/ml LPS at 37°C for another 24 h. The cells lacking LPS and Brevinin-1GHd treatment were regarded as the negative control. The contents of NO, IL-1β, IL-6 and TNF-α in the culture supernatants were, respectively, measured by Griess reagent (Beyotime Biotechnology, China) and ELISA kits (Thermo Fisher Scientific, United States) in light of the manufacturers’ manuals. Nitrite contents reflecting NO production in supernatants were obtained from the standard curve produced with NaNO_2_ (Sigma-Aldrich, St. Louis, Missouri, United States). All experiments were performed with at least three repeats.

### 2.11. Western blot analysis

RAW 264.7 cells were plated in 6-well plates at the number of 1× 10^6^ cells per well overnight for cell adhesion. Then, the macrophages were treated with different concentrations of Brevinin-1GHd (1, 2, and 4 μM) for 30 min, and then co-incubated with 100 ng/ml LPS for another 30 min before collected for WB analysis of phospho-JNK/JNK, phospho-ERK/ERK, phospho-p38/p38 and GAPDH contents (Cell Signaling Technology, Beverly, Massachusetts, USA) as previously described (Zeng et al., 2018). The cells lacking LPS and Brevinin-1GHd treatment were regarded as the negative control. All experiments were undertaken in triplicates.

### 2.12. Paw edema assay

The *in vivo* anti-inflammatory effects of Brevinin-1GHd were evaluated with the carrageenan-induced mice as described previously (Tian et al., 2021). Shortly, at the beginning of the experiment, the paw volume up to the ankle was determined in all mice using a volume meter (Taimeng PV-2007500, China). Then mice were pre-injected with Indometacin (10 mg/kg) or Brevinin-1GHd (5 mg/kg) through intraperitoneal injection. One hour after the administration, the modeling treatment was performed. Except that the right paw of the mice in the control group was treated with 50 ul of normal saline through subcutaneous injection, the other mice were treated with 50 ul of normal saline containing 1% carrageenan in the right paw through subcutaneous injection. Δpaw thickness was calculated from incremental volumes (a–b), where “a” and “b” were the volumes of the mouse’s right hind paw at different time points before and after carrageenan administration. In order to obtain mouse peak right paw tissue, another batch of mice were reprocessed as described above. Four hours after injection of carrageenan, the mouse right hind paws were surgically removed for myeloperoxidase (MPO) activity and histological analysis.

### 2.13. Myeloperoxidase activity assay

Myeloperoxidase (MPO) abundantly express in neutrophil granulocytes, and its presence can directly reflect the content of neutrophil granulocytes. Therefore, MPO kinetic-colorimetric assay was carried out to evaluate the neutrophil migrated to the hind paw. The frozen paws from paw edema assay were homogenated in 600 μl PBS (pH = 7.4), centrifuged at 10,000 rpm/min at 4°C for 15 min to take the supernatant and set it to a uniform concentration. Subsequently, the MPO Detection kit (Nanjing Jiancheng Bioengineering Institute, China) was used to detect the MPO content of each supernatant according to the manual of the kit. The supernatant was added to a 96-well plate pre-filled with PBS solution containing 1% cetyltrimethylammonium, 0.17 mg/ml of 3, 3′-dimethoxybenzidine and 0.0005% H_2_O_2_, and reacted in dark at 37°C for 30 min, then the absorbance values of 460 nm was detected with microplate reader (Tecan Trading AG, Ma¨nnedorf, Switzerland). MPO activity in the tissues was expressed as units of enzyme/g of tissue. One unit of MPO is the amount of enzyme that oxidizes MPO substrate to produce 1 μM luciferin per minute at 37°C.

### 2.14. Statistical analysis

Statistical analysis was carried out using Igor and GraphPad Prism 6.0 software (GraphPad Software Inc., La Jolla, CA, United States). The multiple groups were analyzed using one-way analysis of variance and Tukey’s post-hoc test while the significance between two experimental groups was implemented with unpaired Student’s t-test. All experiments were repeated at least three times and a *p*-value < 0.05 was statistically regarded as significant difference. All the values are shown as mean ± SEM.

## 3. Results

### 3.1. cDNA identification and cladistic analysis

**Figure 1:**
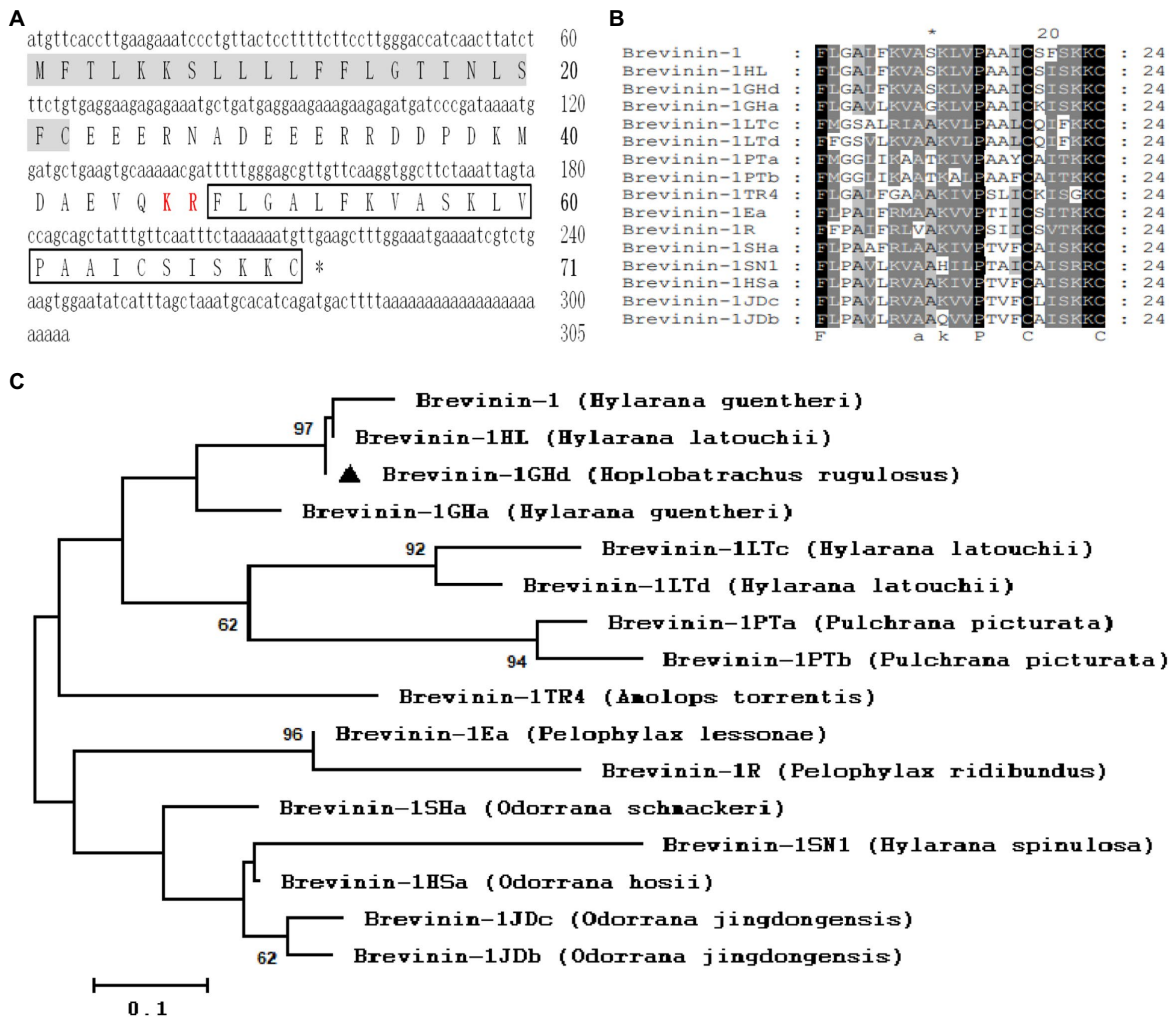
Sequence Analysis of Brevinin-1GHd. **(A)** The cDNA and predicted amino acid sequences of Brevinin-1GHd. The signal peptide and KR protease cleavage site are labeled with a gray background and red style, respectively. The amino acid sequence of mature Brevinin-1GHd is boxed, and the stop codon is indicated by an asterisk (*). **(B)** Amino acid sequence alignment of mature Brevinin-1GHd with other Brevinins-1 peptides from different frogs. The identical residues are marked with a black background. **(C)** Phylogenetic analysis of Brevinins-1 from different frogs. Numbers at the forks symbol the percentage of trees in which this group occurs after bootstrapping (1,000 replicates; shown only when >60%). Scale bar shows the number of substitutions per base. Replicates are applied to prove the reliability of each branch.

The cDNA sequence obtained by the skin transcriptome analysis was 305 bp and encoded a precursor of classic AMP which contained the signal peptide, acidic domain and KR site. BLAST results showed that it was highly identity to Brevinin-1HL and Brevinin-1GHd reported from the skin of *H. latouchii* and *H. guentheri* with the exception of a substitution (Phe^21^ to Leu^21^) in the signal peptide (Jiang et al., 2020; Lin et al., 2021; Figures 1A,B). Thus, the mature peptide sequence was predicted to be FLGALFKVASKLVPAAICSISKKC. The secondary structure model of Brevinin-1GHd predicted by trRosetta was composed of two α-helices, which were composed of nine amino acids near N terminus (Ala^4^-Leu^12^) and five amino acids near C terminus (Ala^15^-Ser^19^), respectively. The predicted helices and coils account for 58.3 and 41.6%, respectively (Supplementary Figure S1). Considering that some biological activities of Brevinin-1GHd have been reported, Brevinin-1GHd was continued to use as the name of the peptide from *H. rugulosus* skin to unify this study with the previous report (Jiang et al., 2020). Like other members of Brevinin-1 family, the deduced peptide contained a “Rana box” structure which was composed by a disulfide bond between two Cys residues at the C terminus (Figures 1A,B). The phylogenetic tree analysis showed that the bootstrapping values among the three was 97, which mean a high affinity existed among these species (Figure 1C).

### 3.2. Circular dichroism determination

**Figure 2:**
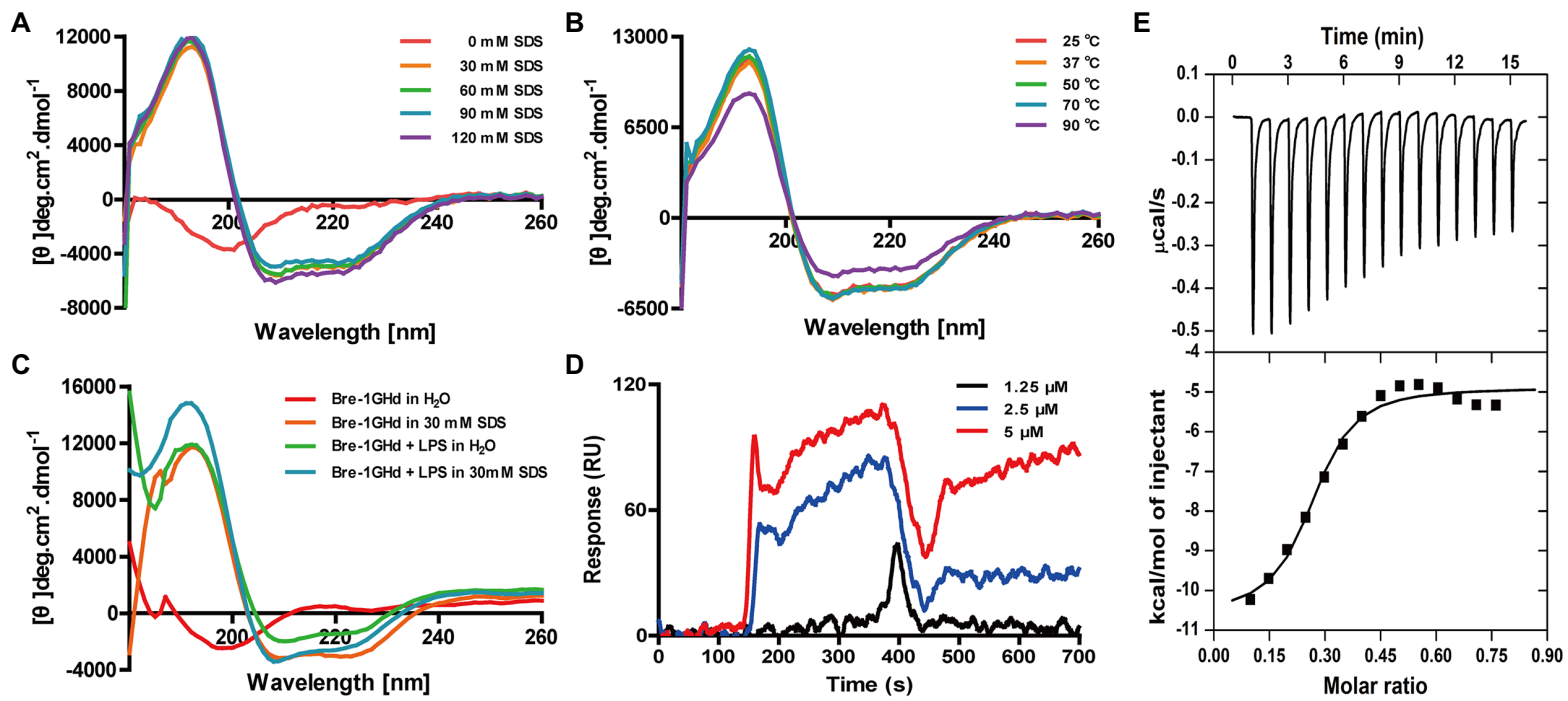
CD analysis of Brevinin-1GHd and binding reaction of Brevinin-1GHd with LPS. **(A–C)** The CD spectra of Brevinin-1GHd in different conditions. **(D)** Representative SPRi sensorgrams of LPS binding to Brevinin-1GHd immobilized on a gold chip. The black, blue, and red lines indicate the SPRi signal generated by 1.25, 2.5, and 5 μM LPS flowing through Brevini-1GHd immobilized on the chip, respectively. **(E)** ITC measurement of Brevinin-1GHd binding to LPS. The top and low panels display thermo changes of each administration and enthalpy changes as a function of ligand/target molar ratio, respectively.

The secondary structure of Brevinin-1GHd was measured by CD determination in different solutions or temperature environments. As showed in Figures 2A,B, the CD curves of Brevinin-1GHd in different concentrations of SDS solutions and under different ambient temperatures were almost the same. Even when the environmental temperature rose to 90°C, the peak of the spectrum decreased slightly while its shape was also basically unchanged, which indicated that Brevinin-1GHd had high stability. In addition, the two negative shoulder band peaks at 222 nm and 208 nm and the positive peak near 192 nm were appear as a characteristic of the α-helical structure of Brevinin-1GHd. By comparing the CD spectra of Brevinin-1GHd after incubation with or without LPS in 30 mM SDS solution, the presence of LPS could rise the peak at 195 nm and the trough at 208 nm on the CD curve of Brevinin-1GHd (Figure 2C), which indicating that LPS bound to the polypeptide and changed the secondary structure of the peptide.

### 3.3. Direct binding reaction with LPS

The binding activity of Brevinin-1GHd to LPS was explored with ITC and SPRi experiments. As showed in Figure 2D, when Brevinin-1GHd was immobilized on the SPRi chip and different concentrations of LPS flowed through the immobilized peptide, strong SPRi signal was appeared and resonance unit increase were concentration-dependent, indicating that LPS could directly bind to Brevinin-1GHd. Furtherly, during the reaction between Brevinin-1GHd and LPS in the ITC reflection pool, the enthalpy decreased and heat was released, indicating that they had combined. In addition, the Kd value of their binding affinity was 6.49 ± 5.40 mM (Figure 2E).

### 3.4. Inhibition of NO and inflammatory cytokines production induced by LPS

**Figure 3:**
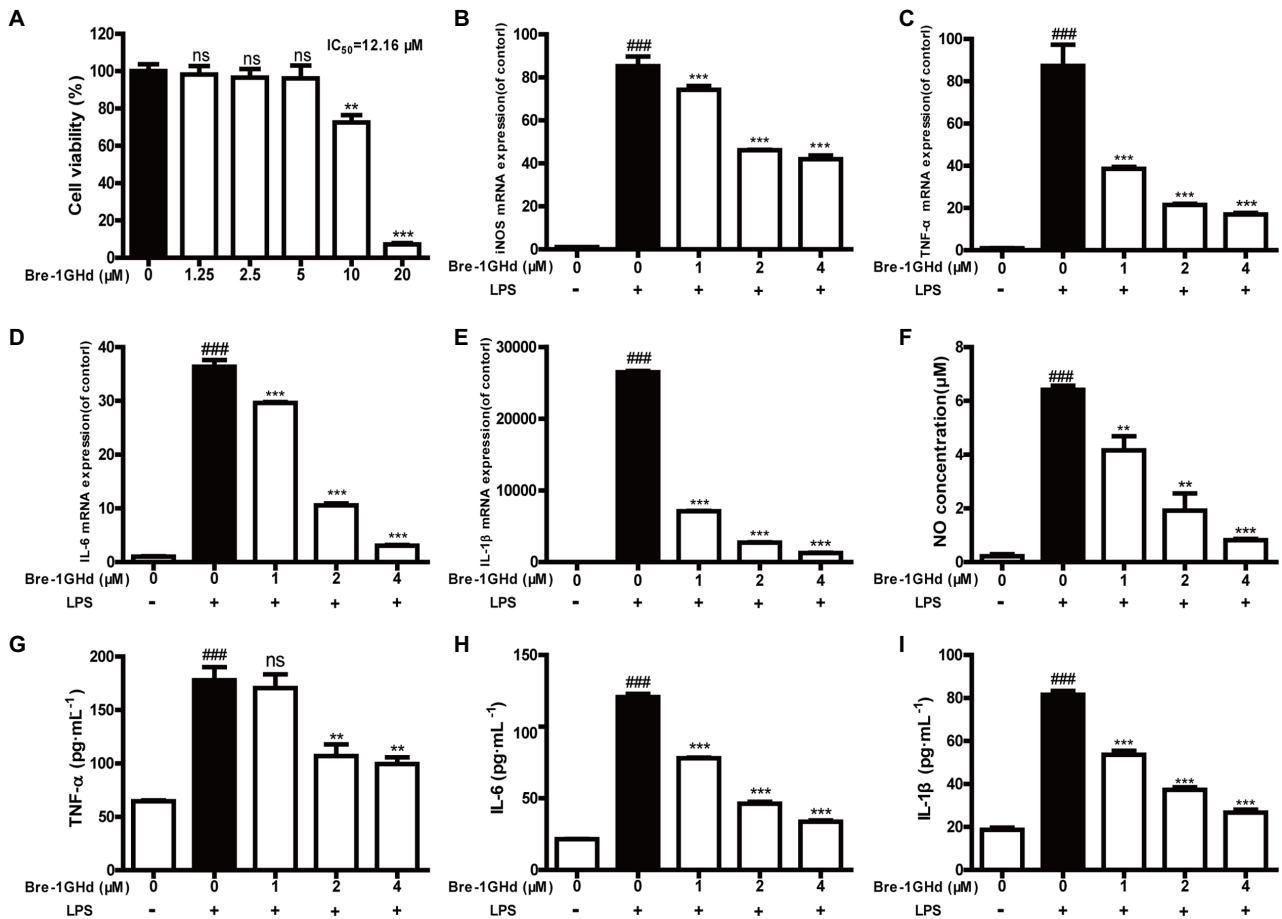
Effects of Brevinin-1GHd on the generation of nitrite and cytokines induced by LPS. **(A)** Proliferation inhibition activity of Brevinin-1GHd on RAW 264.7 cells. The cells were incubated with Brevinin-1GHd (1.25–20 μM) for 24 h, and then the IC_50_ value of Brevinin-1GHd on RAW264.7 cells was determined by CCK-8. **(B–I)** The effect of Brevinin-1GHd on production of NO, TNF-α, IL-6 and IL-1β in RAW 264.7 cells recruited with LPS. Data are mean ± SEM (*n* = 3). ^∗∗^*p* < 0.01 and ^∗∗∗^ *p* < 0.001 as well as ^###^ *p* < 0.001 indicate significant difference in comparison with the control incubated with 100 ng/ml LPS and the control without LPS plus Brevinin-1GHd, respectively.

It is well known that LPS can induce the inflammatory action of macrophages (Luo and Song, 2021). The anti-inflammatory activity of Brevinin-1GHd was thus tested in LPS-induced mouse macrophages. Firstly, the proliferation inhibition activity of Brevinin-1GHd was tested on RAW 264.7 cells by the CCK-8 method to ensure that the concentrations used in subsequent experiments would not affect cell viability. As shown by Figure 3A, the IC_50_ value of Brevinin-1GHd on RAW 264.7 cells was about 12.16 μM (Figure 3A). Subsequently, the mRNA expression levels of four inflammatory factors were measured in RAW 264.7 cells by real-time PCR. After LPS stimulation, the mRNA levels of iNOS, TNF-α, IL-6 and IL-1β in RAW 264.7 cells were significantly increased while Brevinin-1GHd could concentration-dependently inhibit the stimulation effect of LPS. In the group pretreated with 4 μM Brevinin-1GHd for 30 min, the expression levels of iNOS, TNF-α, IL-6 and IL-1β mRNA were reduced by about 50.69, 80.58, 91.61 and 95.19% compared to the LPS-stimulated group, respectively (Figures 3B–E). Similar to the results of real-time PCR, RAW 264.7 cells stimulated by LPS released a large amount of NO, TNF-α, IL-6 and IL-1β, and these effects were significantly suppressed by Brevinin-1GHd in a concentration-dependent manner. As showed in Figures 3F–I, after treatment with 4 μM Brevinin-1GHd, the release of NO, TNF-α, IL-6 and IL-1β were reduced by about 87.31, 44.09, 72.10 and 67.20%, respectively. Thus, Brevinin-1GHd could significantly suppress the transcription and expression of inflammatory factors in RAW 264.7 cells stimulated by LPS at a concentration that was non-toxic to cells.

### 3.5. Suppression of LPS-induced inflammatory response pathways

**Figure 4:**
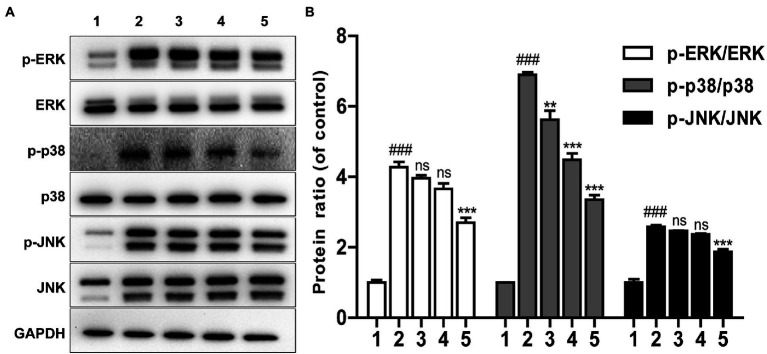
Effects of Brevinin-1GHd on inflammatory pathway activation inspired by LPS. **(A)** Typical WB bands of ERK, p38, JNK in RAW 264.7 cells. The cells were pre-treated with or without 1, 2, and 4 μM Brevinin-1GHd for 30 min and an additional 30 min after LPS addition, then were collected for WB analysis. ICONS 1–5 are the control group, LPS treatment group, and 100 ng/ml LPS plus 1, 2, and 4 μM Brevinin-1GHd treatment groups, respectively. **(B)** Quantification analysis of band densities in A figure. Data are expressed as mean ± SEM (*n* = 3). ^∗∗^*p* < 0.01 and ^∗∗∗^*p* < 0.001 as well as ^###^ *p* < 0.001 suggest significant difference in comparison with the control incubated with 100 ng/ml LPS and with the control without both LPS and Brevinin-1GHd, respectively.

It is well known that LPS can induce the phosphorylation of JNK, ERK and p38 protein which are main components of MAPK pathway (Negishi et al., 2006; Cao et al., 2019). The effect of Brevinin-1GHd was thus investigated on the inflammation-related signaling pathways of RAW 264.7 cells activated by LPS with WB assays. As presented in Figure 4A, compared with the control without LPS stimulation, the expressions of phosphorylated JNK, ERK and p38 in the LPS stimulation group were increased significantly, which indicated that LPS successfully activated the MAPK pathway of RAW 264.7 cells. Meanwhile, this effect of LPS was significantly reduced by Brevinin-1GHd in a concentration-dependent manner. The ratios of p-JNK/JNK, p-ERK/ERK and p-p38/p38 in the 4 μM Brevinin-1GHd group were reduced by about 27.68, 36.80 and 51.29%, respectively, compared with the LPS-stimulated group (Figure 4B). This result indicated that Brevinin-1GHd mainly acted on the MAPK signaling pathway of RAW 264.7 cells, thereby inhibiting the inflammatory response induced by LPS.

### 3.6. Paw edema assay

**Figure 5:**
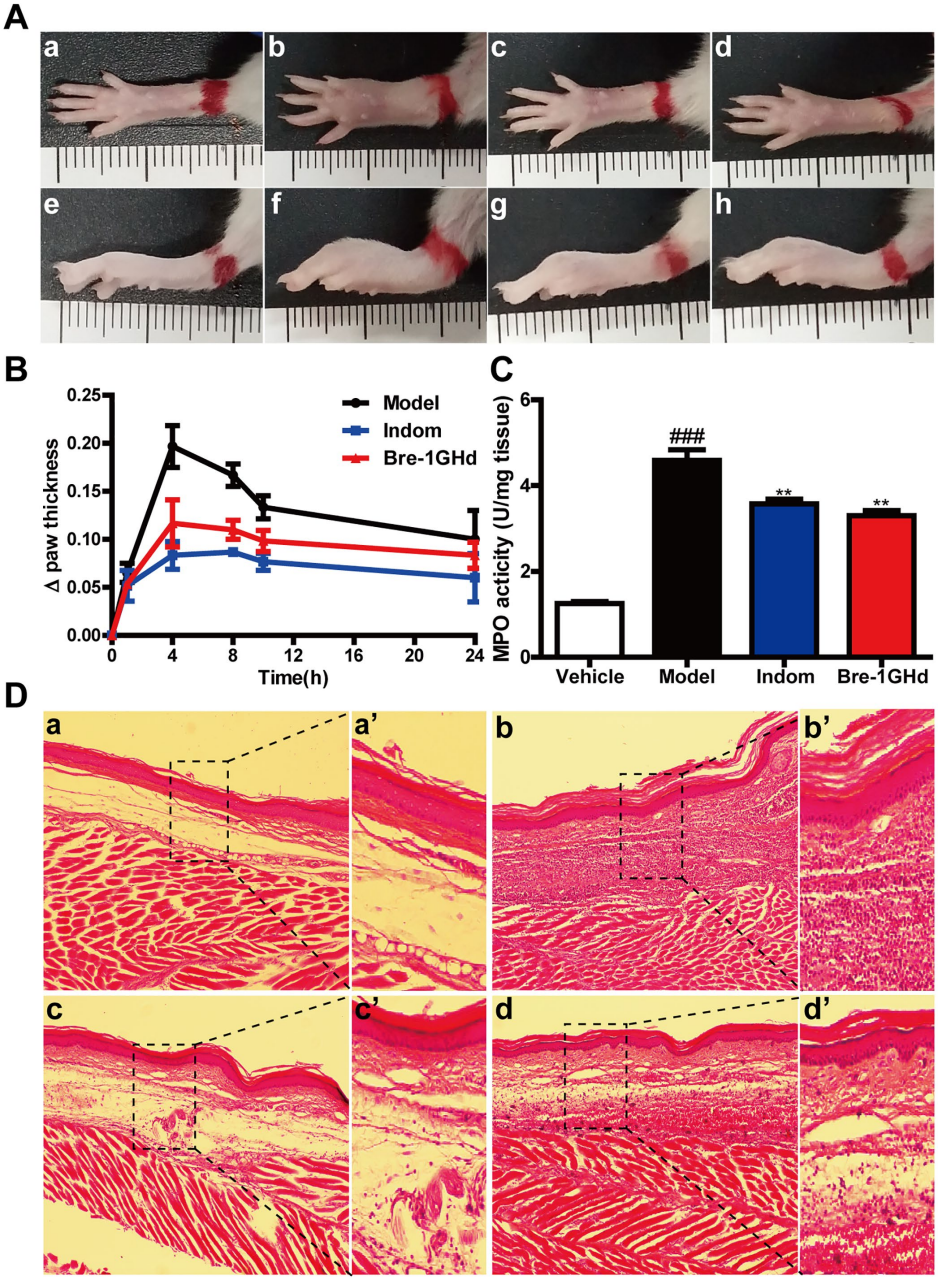
Treatment of carrageenan-induced acute inflammation in mice paw by Brevinin-1GHd. **(A)** Mice paws at 4 h after carrageenan administration. Panels **a–d** and **e–h** indicate the corresponding bottom and lateral views of the paws. Images from left to right are the control group **(a,e)**, the model group **(b,f)**, Indomethacin group **(c,g)** and Brevinin-1GHd group **(d,h)**. **(B)** Thickness of right paw of mice at different time points within 24 h. **(C)** MPO activity in the right paw of mice at 4 h after administration. **(D)** HE staining of the right paw of mice at 4 h after administration. Labels **(a,a’)**–**(d,d’)** refer to the control group, model group, Indomethacin group and Brevinin-1GHd group, respectively. The scale bars of a-d and a’-d’ were 50 and 200 μm, respectively.

Carrageenan-induced mice paw edema assay was carried out to study the anti-inflammatory effects of Brevinin-1GHd *in vivo* (Park and Im, 2021). As shown in Figure 5A, carrageenan administration obviously promoted the development of the inflammatory vascular phase of the right paw of mice, resulting in increased skin edema on the hind paw, however, intraperitoneal injection of 10 mg/kg Indomethacin or 5 mg/kg Brevinin-1GHd into mice before carrageenan injection could significantly alleviate the swelling of the right paw of mice. The measurement results showed that the swelling volume of the right paw treated by Brevinin-1GHd and Indomethacin at 4 h was reduced by about 57.62 and 40.67%, respectively, compared with the mice in the model group and the swelling volume of the right paw in 24 h was also reduced by about 40.00 and 16.66%, respectively (Figure 5B). In addition, MPO levels in the right paw tissue grinding fluid of mice injected with carrageenan increased dramatically. Compared with the mice in the model group injected with carrageenan alone, the MPO concentration in the right paw tissue grinding fluid of mice injected with Indomethacin or Brevinin-1GHd before carrageenan injection decreased by about 20.03 and 27.98%, respectively (Figure 5C). Furthermore, histopathological evaluation showed that the paw tissue of mice in the model group only exposed to carrageenan had epithelial hyperplasia, inflammatory cell infiltration and sub-epidermal edema, while Indomethacin and Brevinin-1GHd significantly prevented these signs of inflammation (Figure 5D). The above data suggested that Brevinin-1GHd had a significant inhibitory effect on the development of acute edema in the right paw of mice.

## 4. Discussion and conclusions

The increasing number of AMPs have been demonstrated to show both antimicrobial and immunomodulatory activities due to their unique physicochemical properties and mechanism of action, which make them have great potential in the treatment of infectious inflammation (Mahlapuu et al., 2016; Luo and Song, 2021). The immunoregulatory activity of Brevinin-1 family, however, has not been reported so far. In this study, Brevinin-1GHd was proved to possess LPS neutralizing as well as anti-inflammatory activity *in vitro* and *in vivo*.

The evolution of AMPs in amphibians is characterized by frequent gene duplication and diversification driven by positive selection, which can cause the generation of resistance genes with new functional properties. Furthermore, functionally divergent AMPs can cope with new emerging pathogens in the environment or old pathogens that have developed resistance (Lazzaro et al., 2020). As a result, it makes the molecular variation within a particular AMP family is extremely large, meaning a peptide from one species little show an identical amino acid sequence in another, even when two species are quite close in the evolutionary relationship (Tennessen and Blouin, 2007). For the members belonging to Brevinin-1 family widely identified in most Eurasian and New World species, the discovery of Brevinin-1 peptides with the same amino acid sequence in different species occurs occasionally (Savelyeva et al., 2014). In this study, a peptide identified from the skin of *H. rugulosus* frog is with complete identity to known AMP in *H. guentheri* and *H. latouchi*. The repeated expression of Brevinin-1GHd in the three species may be an important basis for their common evolutionary origin. After all, it has been reported that *Hoplobatrachus* and *Hylarana* species are most likely originated from southern Asia (Sultana et al., 2017; Reilly et al., 2022). In addition, *H. rugulosus* is mainly distributed in southern China and broadly inhabits rice fields, pools or stone crevices, which are similar to *H. guentheri* and *H. latouchi*. The resembling geographical environment is probably one of the key factors to maintain conservative expression of Brevinin-1GHd in three species during the evolutionary process (AmphibiaChina, 2022). Furthermore, it is possible that other AMPs like Brevinin-1GHd are co-expressed in different species, and the reason why these AMPs remain in the process of different species evolution are worth further being explored.

LPS is a momentous component in the cell wall of Gram-negative bacteria (Zhang et al., 2013) and often released into the blood circulation during infection, inducing monocytes and phagocytes to produce a large amount of cytokines and local inflammation (Maldonado et al., 2016). Therefore, the neutralization of LPS produced by bacteria is the key to the treatment of infectious inflammation. Coincidentally, most AMPs are positive charges and have a high content of hydrophobic residues. Positive charges can promote strong electrostatic interaction between AMPs and negatively charged LPS, and hydrophobicity makes them easy to be embedded in LPS micelles. Thus, some AMPs have the ability to bind LPS (Sun and Shang, 2015; Schromm et al., 2021). Considering that Brevinin-1GHd contains not only four positively charged lysine residues, but also hydrophobic amino acids such as alanine, leucine, valine, proline, isoleucine and phenylalanine, it was speculated to have LPS-neutralizing activity. In agreement, the overwhelming evidences from ITC, SPRi, CD and WB experiments demonstrated this speculation. Interestingly, although the secondary structures of Brevinin-1GHd and Cath-MH are very similar and both dominated by α-helices with short random coils at both ends, the LPS-neutralization ability of Brevinin-1GHd is significantly weaker than that of Cath-MH (Chai et al., 2021). By comparing their sequences and physicochemical properties, Cath-MH is found to contain more positively charged residues than Brevinin-1GHd. Furtherly, Brevinin-1GHd showed more significant anti-inflammatory activity than Brevinin-2MP in the treatment of carrageenan-induced acute inflammation in animal models when comparing present results with data reported by Tian (Tian et al., 2021). Although Brevinin-2MP has one more cation (Arg) than Brevinin-1GHd, it also has two more negatively charged residuals (Asp + Glu). This may prove that cationic property is one of the keys to affect the anti-inflammatory activity of AMPs (Sun and Shang, 2015). Beyond all doubt, such inferences need be verified in future experiments.

It is worth noting that Brevinin-1GHd also shows good anti-inflammatory activity in carrageenan-induced mouse paw swelling which lacks LPS (Figure 5). Some AMPs have been described to exert their anti-inflammatory activities by directly acting on cell surface receptors. For example, PS-K18 can inhibit TLR4-mediated NF-κB pathway and activate innate defense (Jang et al., 2019). In addition, Brevinin-1GHd could bind cell surface in a concentration dependent manner which indicate that it can also directly bind to some receptors on RAW 264.7 cells (Supplementary Figure S4). Therefore, the anti-inflammatory mechanism of Brevinin-1GHd maybe be also related to its receptors on the cell surface and further study is necessary to clarify its anti-inflammatory mechanism completely.

In conclusion, including broad-spectrum antibacterial activity, Brevinin-1GHd can not only neutralize LPS rapidly, but also effectively inhibit the inflammation of RAW 264.7 cells induced by LPS. At the same time, Brevinin-1GHd also shows significant therapeutic effect on carrageenan-induced acute inflammation of mice paws. These biological activities make Brevinin-1GHd very likely to be a new anti-infective agent to assist or replace antibiotics.

## Data availability statement

The original contributions presented in the study are included in the article/Supplementary material, further inquiries can be directed to the corresponding authors.

## Author contributions

MT, KW, YL, JC, JW, HZ, and XH performed experiments and analyzed data. XC and XX designed experiments, supervised the study, evaluated the data, and wrote and revised the manuscript for publication. All authors contributed to the article and approved the submitted version.

## Funding

This study was supported in part by the National Natural Science Foundation of China (Nos, 31772476, 31911530077, 31861143050, and 82000040).

## Conflict of interest

The authors declare that the research was conducted in the absence of any commercial or financial relationships that could be construed as a potential conflict of interest.

## Publisher’s note

All claims expressed in this article are solely those of the authors and do not necessarily represent those of their affiliated organizations, or those of the publisher, the editors and the reviewers. Any product that may be evaluated in this article, or claim that may be made by its manufacturer, is not guaranteed or endorsed by the publisher.

## Supplementary material

The Supplementary material for this article can be found online at: https://www.frontiersin.org/articles/10.3389/fmicb.2023.1102576/full#supplementary-material

## Footnotes

^1^ https://web.expasy.org/protparam/

^2^ https://www.cbs.dtu.dk/services/SignalP/

^3^ https://yanglab.nankai.edu.cn/trRosetta/

^4^ www.megasoftware.net
