# Supplementary material for: The first Brevinin-1 antimicrobial peptide with LPS-neutralizing and anti-inflammatory activities in vitro and in vivo
Source: Front Microbiol. 2023 Mar 3;14:1102576. doi: 10.3389/fmicb.2023.1102576 (PMC10020232; doi:10.3389/fmicb.2023.1102576)
Supplement: Supplementary file 1 [file Data_Sheet_1.docx]

Supplementary Material

**Supplementary Figure 1.** Prediction of secondary structure of Brevinin-1GHd. (A) Secondary structure modeling of Brevinin-1GHd produced by trRosetta and visualized with PyMOL. The predicted Brevinin-1GHd structure was shown in the form of ribbons, with blue, gray and yellow indicating helix, coil and disulfide bond respectively. The positively charged amino acids and Cysteines were labeled. (B) Predicted information of Brevinin-1GHd from trRosetta.

**Supplementary Figure 2.** Brevinin-1GHd was purified and identified by HPLC.

**Supplementary Figure 3.** The purity and identity of Brevinin-1GHd was confirmed by MALDI-TOF-MS.

**Supplementary Figure 4.** The binding of Brevinin-1GHd to mouse macrophage. FITC-labeled Brevinin-1GHd (1.25 2.5, 5, and 10 μM) was incubated with RAW 264.7 cells for 30 min before flow cytometry.

| **Name** | **5’ primer** | **3’ primer** |
| --- | --- | --- |
| iNOS | 5’ -CTGCAGCACTTGGATCAGGAACCTG-3’ | 5’ GGAGTAGCCTGTGTGCACCTGGAA-3’ |
| TNF-α | 5’ -CGGTGCCTATGTCTCAGCCT- 3’ | 5’ –GAGGGTCTGGGCCATAGAAC-3’ |
| IL-6 | 5’- AGTTGCCTTCTTGGGACTGA-3’ | 5’- TCCACGATTTCCCAGAGAAC-3’ |
| IL-1β | 5’ -ATGGCAACTGTTCCTGAACTC3’ | 5’- GCCCATACTTTAGGAAGACA-3’ |
| GAPDH | 5’ –GTGAAGGTCGGTGTGAACGGATT-3’ | 5’- GGAGATGATGACCCTTTTGGCTC-3’ |

**Supplementary Table 1.** Primers (mouse) used for qRT-PCR
