# Supplementary figures and images for: The first Brevinin-1 antimicrobial peptide with LPS-neutralizing and anti-inflammatory activities in vitro and in vivo
Source: Front Microbiol. 2023 Mar 3;14:1102576. doi: 10.3389/fmicb.2023.1102576 (PMC10020232; doi:10.3389/fmicb.2023.1102576)

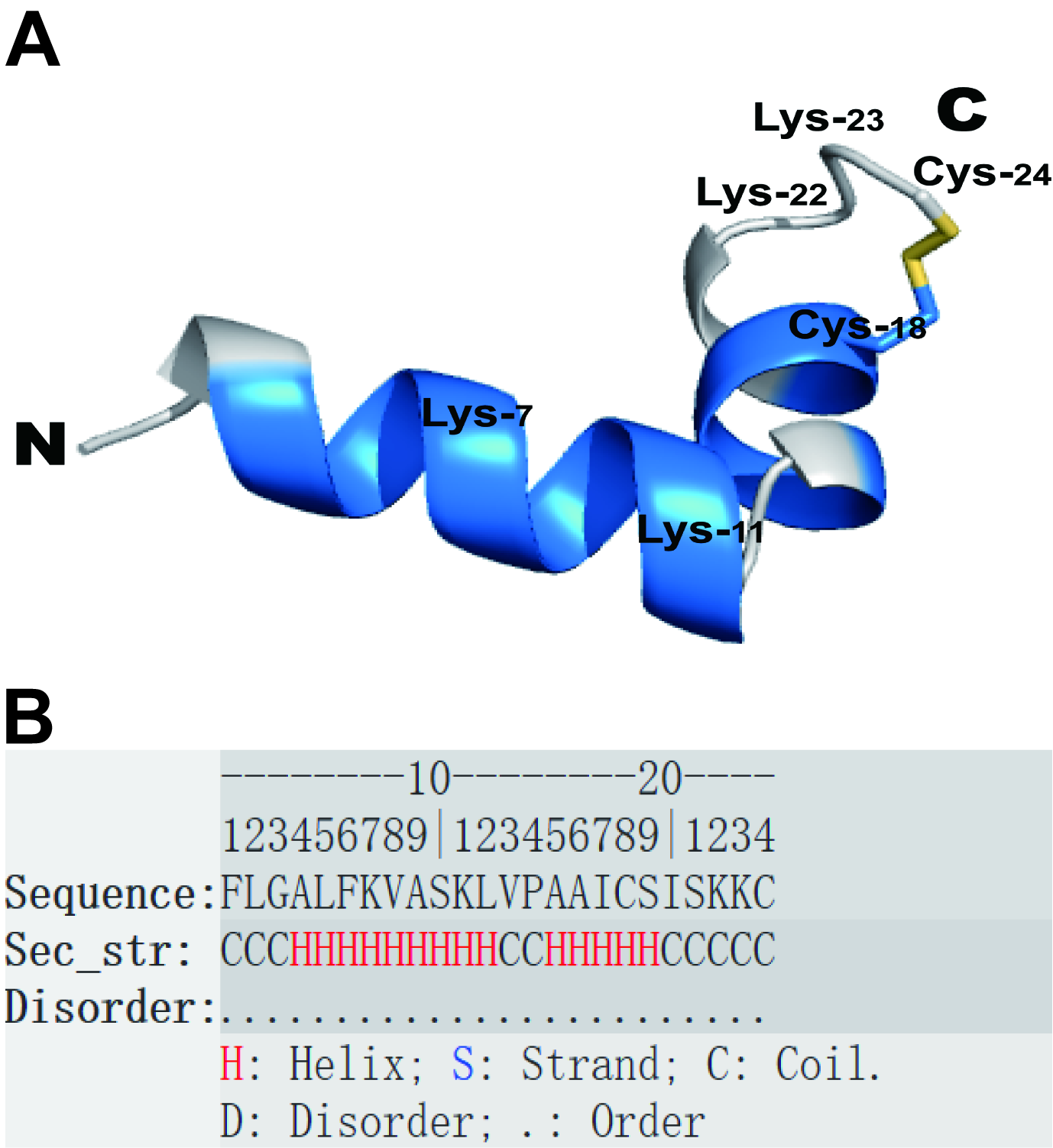

Supplement: Supplementary file 2 [file Image_1.TIF]

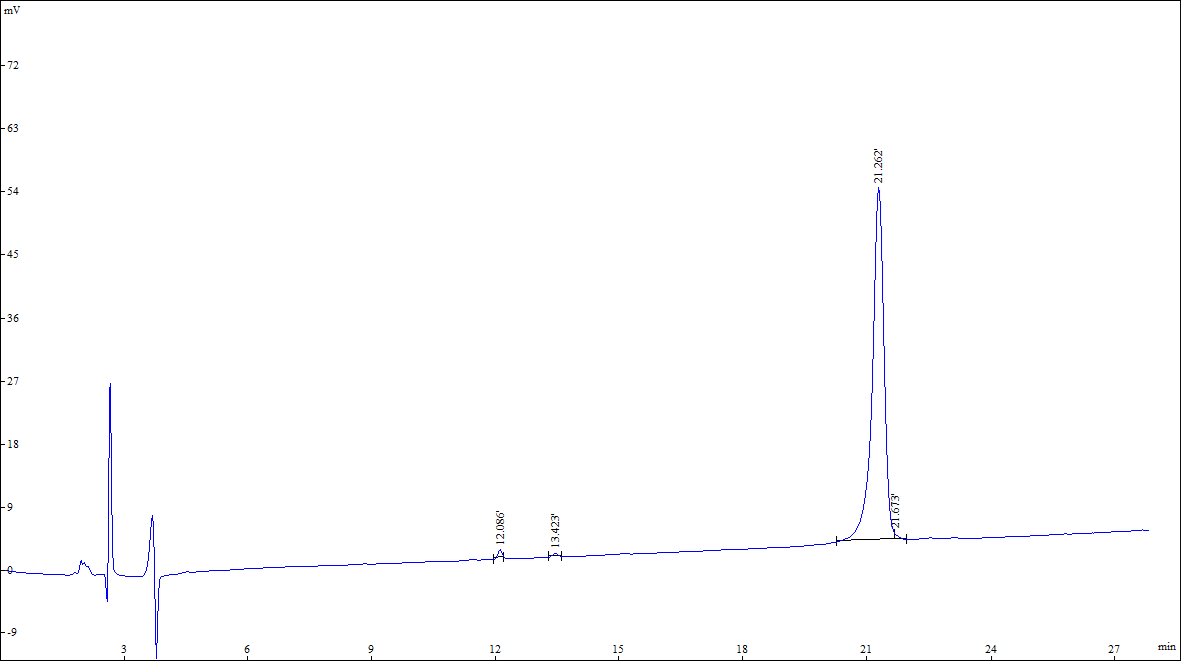

Supplement: Supplementary file 3 [file Image_2.TIF]

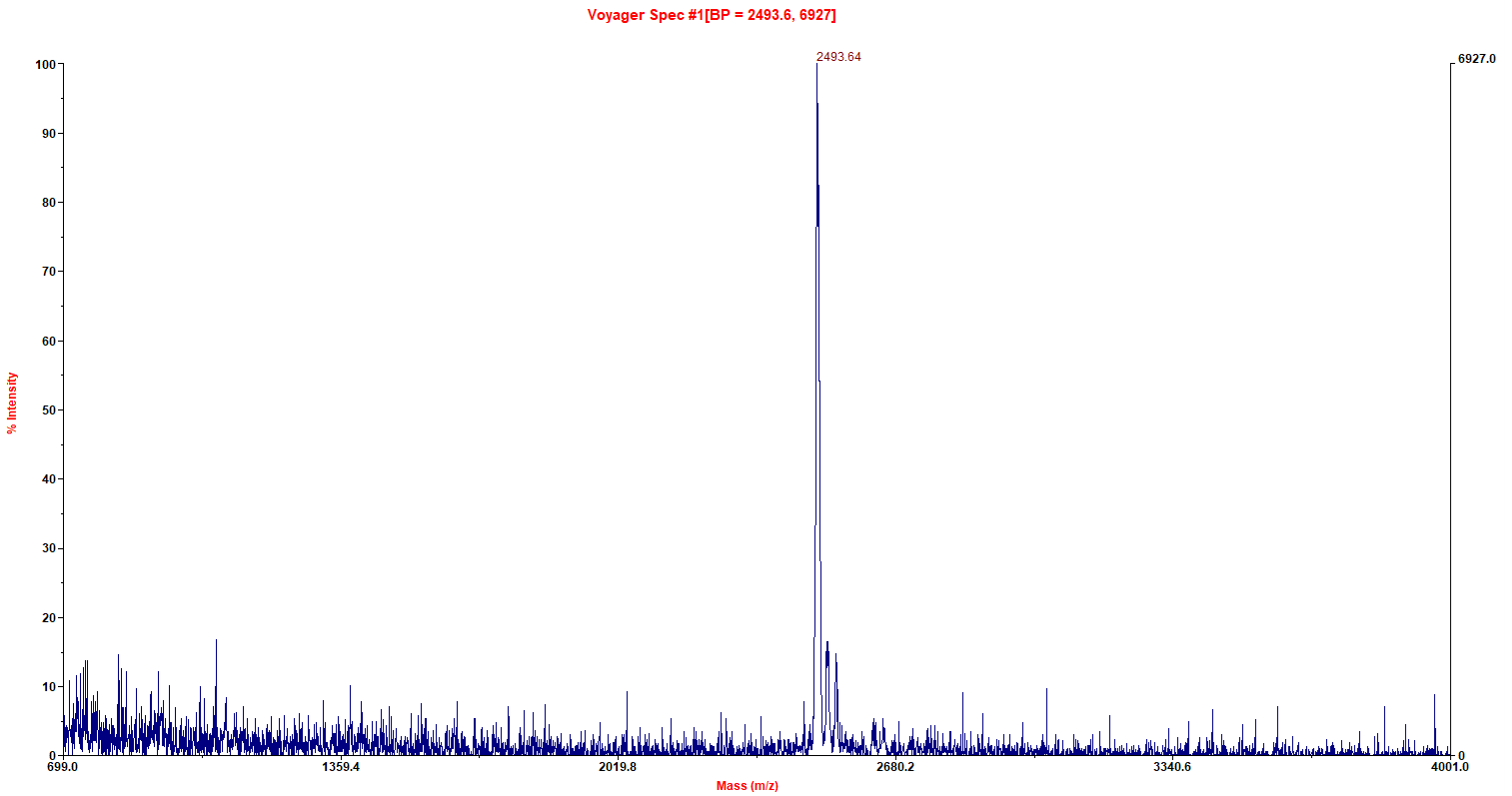

Supplement: Supplementary file 4 [file Image_3.TIF]

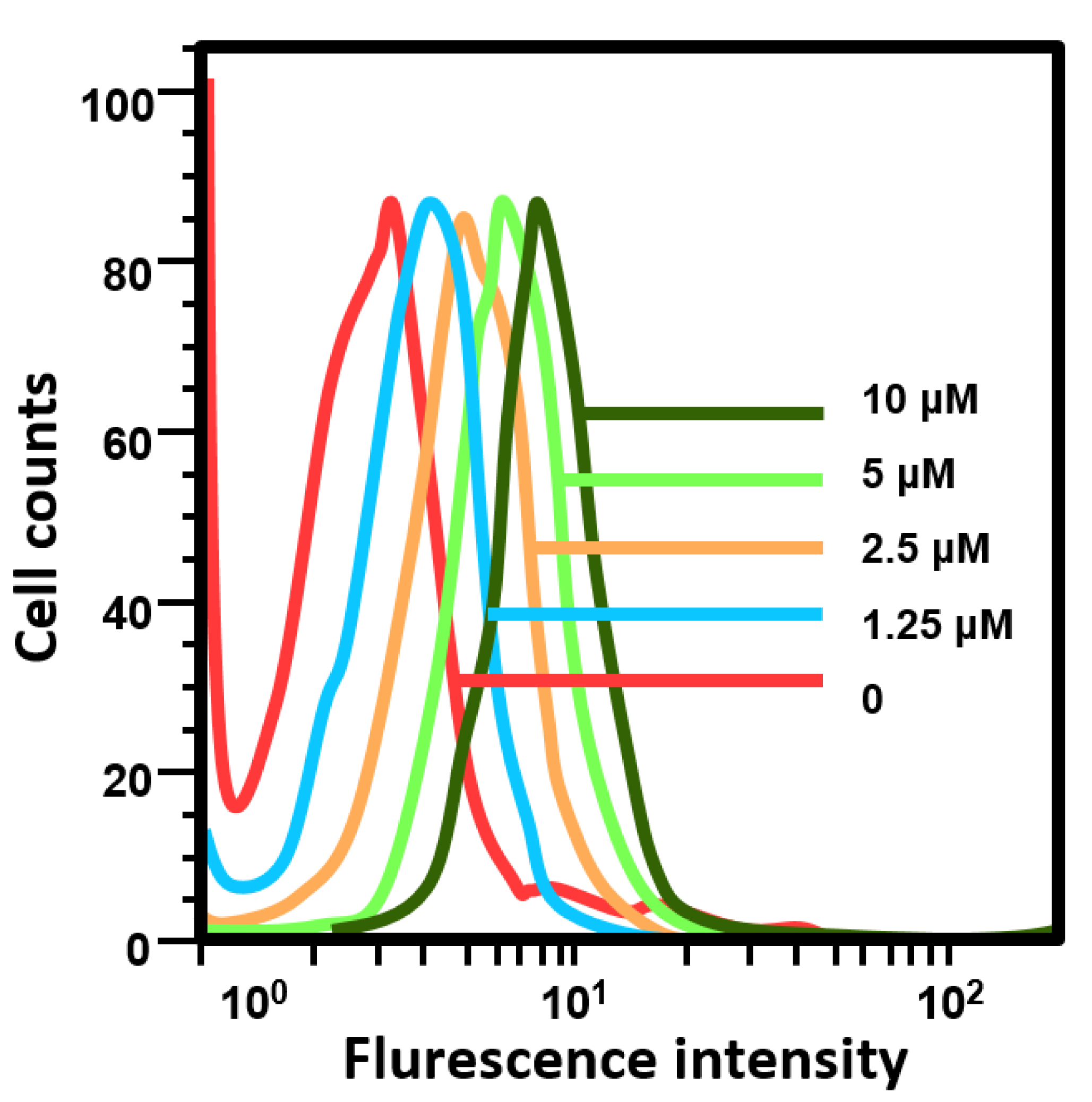

Supplement: Supplementary file 5 [file Image_4.TIF]
